# Survey on Attitudes of Social Engagement

**DOI:** 10.1192/bjo.2026.11085

**Published:** 2026-06-30

**Authors:** Nismen Lathif, Esha Lathif

**Affiliations:** 1 Merseycare NHS Trust, Liverpool, United Kingdom; 2 Blue Coat School, Liverpool, United Kingdom

## Abstract

**Aims:**

Attitudes toward social engagement describe how individuals perceive, value, and participate in social interactions and communal activities. Currently upcoming trends such as Fear of missing out is governing social interactions but one may wonder are there accompanying trends such as Joy and Relief of missing out. Attitudes toward social engagement reflect how people navigate the tension between connection and independence and in this study, we aim to evaluate these attitudes based on Fear/Joy/Relief of missing out on social obligations. An exploration of balance between importance of internal values versus social acceptance is also explored.

**Methods:**

A questionnaire on how social engagement is perceived by an individual was developed based on principles of anxiety feelings of being left out within social events compared to relief of missing social engagement and to satisfaction that comes from choosing not to participate in certain activities.

Alongside generic questions on autonomy as compared to social pressures were evaluated within survey.

There were 92 respondents on an online survey.

**Results:**

67% responders reported that they have moderate social interaction.

30% responders reported that they are affected by pressures of missing out.

Less than 20% responders reported comparison with others’ experiences.

15% reported distress on seeing others’ activities.

50–60% responders reported preference for solitude, time for oneself and own schedule.

45% responders reported satisfaction if they could decline obligations.

40% responders reported relief when they are excluded from social obligations.

45% responders reported relief if social plans fail.

There was evidence of empowerment when avoiding social media.

75% responders reported importance of internal values as opposed to social obligations.

Though 35% felt need to be a part of social group there was limited evidence on need for acceptance and even lesser on comparing with others.

**Conclusion:**

Though Fear of missing out (FOMO) is an upcoming social trend within community interactions a generic survey indicated that a significant majority feel the need for importance of internal values, solitude and preference for time for individual activities as opposed to social obligations. Interestingly higher number of responders reported relief if excluded from social obligations and greater satisfaction if they could decline obligations. The survey also showed less importance for external validation from social circles with limited need for acceptance and even limited comparison with others’ activities. It may be interesting to note that a significant majority of responders felt a greater relief on missing outon social activities rather than the fear of missing out. One may wonder whether the Fear of missing out is overemphasized whilst in actuality there is relief or joy of being excluded.

